# Systematic review and meta-analysis of the effectiveness of chatbots on lifestyle behaviours

**DOI:** 10.1038/s41746-023-00856-1

**Published:** 2023-06-23

**Authors:** Ben Singh, Timothy Olds, Jacinta Brinsley, Dot Dumuid, Rosa Virgara, Lisa Matricciani, Amanda Watson, Kimberley Szeto, Emily Eglitis, Aaron Miatke, Catherine E. M. Simpson, Corneel Vandelanotte, Carol Maher

**Affiliations:** 1 Alliance for Research in Exercise Nutrition and Activity (ARENA), University of South Australia, Adelaide, SA Australia; 2 School of Health, Medical and Applied Sciences, Central Queensland University, Rockhampton, QLD Australia

**Keywords:** Lifestyle modification, Preventive medicine

## Abstract

Chatbots (also known as conversational agents and virtual assistants) offer the potential to deliver healthcare in an efficient, appealing and personalised manner. The purpose of this systematic review and meta-analysis was to evaluate the efficacy of chatbot interventions designed to improve physical activity, diet and sleep. Electronic databases were searched for randomised and non-randomised controlled trials, and pre-post trials that evaluated chatbot interventions targeting physical activity, diet and/or sleep, published before 1 September 2022. Outcomes were total physical activity, steps, moderate-to-vigorous physical activity (MVPA), fruit and vegetable consumption, sleep quality and sleep duration. Standardised mean differences (SMD) were calculated to compare intervention effects. Subgroup analyses were conducted to assess chatbot type, intervention type, duration, output and use of artificial intelligence. Risk of bias was assessed using the Effective Public Health Practice Project Quality Assessment tool. Nineteen trials were included. Sample sizes ranged between 25–958, and mean participant age ranged between 9–71 years. Most interventions (*n* = 15, 79%) targeted physical activity, and most trials had a low-quality rating (*n* = 14, 74%). Meta-analysis results showed significant effects (all *p* < 0.05) of chatbots for increasing total physical activity (SMD = 0.28 [95% CI = 0.16, 0.40]), daily steps (SMD = 0.28 [95% CI = 0.17, 0.39]), MVPA (SMD = 0.53 [95% CI = 0.24, 0.83]), fruit and vegetable consumption (SMD = 0.59 [95% CI = 0.25, 0.93]), sleep duration (SMD = 0.44 [95% CI = 0.32, 0.55]) and sleep quality (SMD = 0.50 [95% CI = 0.09, 0.90]). Subgroup analyses showed that text-based, and artificial intelligence chatbots were more efficacious than speech/voice chatbots for fruit and vegetable consumption, and multicomponent interventions were more efficacious than chatbot-only interventions for sleep duration and sleep quality (all *p* < 0.05). Findings from this systematic review and meta-analysis indicate that chatbot interventions are efficacious for increasing physical activity, fruit and vegetable consumption, sleep duration and sleep quality. Chatbot interventions were efficacious across a range of populations and age groups, with both short- and longer-term interventions, and chatbot only and multicomponent interventions being efficacious.

## Introduction

Insufficient physical activity, excessive sedentary behaviour, poor diet and poor sleep are major global health issues and are among the leading modifiable causes of depression, anxiety and chronic diseases including type 2 diabetes, cardiovascular disease, obesity, cancers and increased mortality^1–3^. The global economic burden of chronic diseases has been estimated to be $47 trillion (USD) between 2010 and 2025^4^. Programs to assist people to adopt healthier lifestyles to prevent and delay the onset of chronic diseases are urgently needed.

Findings from previous systematic reviews and meta-analyses show that various forms of interventions are effective for improving physical activity, diet and sleep^5–11^. Receiving personalised support from health professionals, such as general practitioners/physicians, dietitians and exercise physiologists, is one of the most effective interventions to improve these behaviours. However, interaction with health professionals often requires traditional on-site (in-person) visits, and substantial time, travel and financial costs for patients^12^. Furthermore, these services are often limited to specific patient populations, such as those with a diagnosed chronic disease. As such, many individuals with poor health behaviours (who are at increased risk of chronic disease), may have limited access to support from health professionals to modify their lifestyle and reduce disease risk in the future. Overall, changing these behaviours requires sustained intervention, which can be cost-, time- and resource-intensive^13^. Therefore, cost-effective and feasible behaviour change interventions are required to reduce the prevalence of physical inactivity, poor diet and poor sleep.

Advances in technology and increased access to the internet, and devices such as smartphones and computers has offered new opportunities to deliver accessible, individualised, and cost-effective behaviour change interventions. Previously evaluated online and digital-based interventions targeted towards improving physical activity, sleep and healthy eating have shown to be effective^14,15^, yet several studies have highlighted various challenges including a lack of initial and sustained engagement of users, poor long-term adherence, attrition, and a lack of ability of the intervention to adapt to the changing needs of participants^16–19^.

Chatbots are conversational agents that act to replicate human interaction through text, speech, and visual forms of communication^20,21^. Some chatbots use artificial intelligence (AI) and can be programmed with scripted conversations, questions, and the ability to provide individualised responses based on input from the user. Artificial intelligence, including machine learning (a statistical process of training models with data to make predictions based on a variety of features) and natural language processing (NLP; the ability to identify and analyse verbal and written language) can be used to interact with users via voice, text, and other inputs and outputs^22,23^. Chatbots offer the potential to provide accessible, autonomous, and engaging health-related information and services, and have great potential to increase the accessibility and efficacy of individualised lifestyle modification interventions^24,25^. Previous findings indicate that chatbot interventions are effective for improving depression, anxiety, stress, medication adherence^21,25,26^, and smoking cessation and reducing substance abuse^27^. While previous reviews which have evaluated the effectiveness of chatbot interventions for improving health behaviours^20,28^, including physical activity^29,30^ and diet^30^ have provided preliminary support for chatbot interventions, but have not involved meta-analyses. Therefore, the purpose of this systematic review and meta-analysis was to evaluate the efficacy of chatbot interventions designed to improve physical activity, diet and sleep.

The key findings of our study are that chatbot interventions targeting physical activity, fruit and vegetable consumption, sleep duration, and sleep quality show significant effects in improving these outcomes. Text-based and AI chatbots are more effective than speech/voice chatbots for promoting fruit and vegetable consumption, while multicomponent interventions are more effective for improving sleep duration and quality. Overall, chatbot interventions are effective across populations and age groups, with varying intervention durations and components.

## Results

### Literature search and screening

**Fig. 1:**
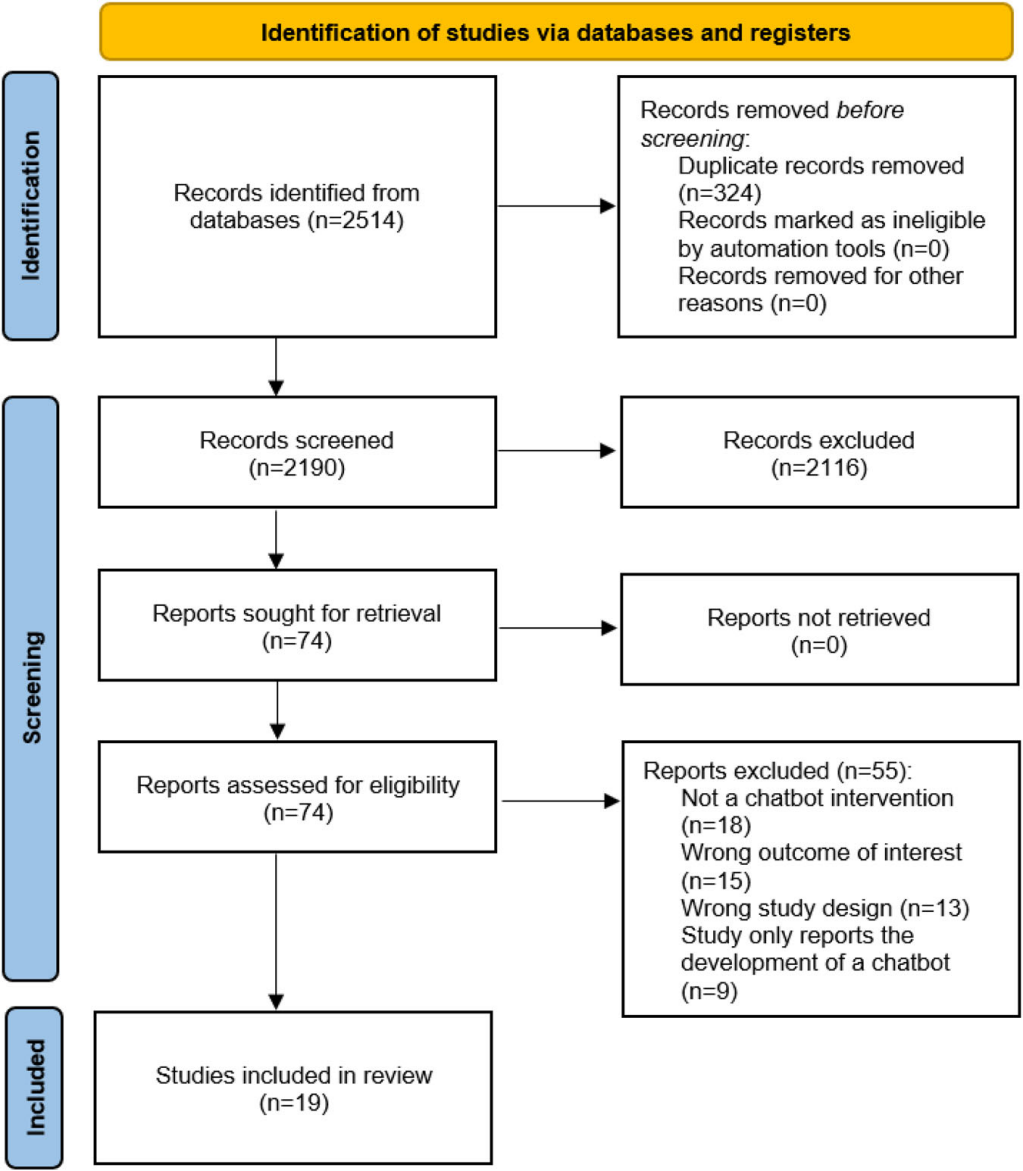
PRISMA flow diagram showing the study selection process.

After a search of databases, 2514 records were identified (see Fig. 1 for PRISMA flow diagram). Following removal of duplicates, and title and abstract screening, 74 full texts were retrieved, of which 19 were included (see Fig. 1 for reasons for exclusions). The included studies comprised of 11 RCTs, 2 non-RTs, 5 single group pre-post studies, and 1 panel design trial. An overview of risk of bias ratings based on the Effective Public Health Practice Project Quality Assessment (EPHPP) tool is shown in Supplementary Table 1. Most studies (*n* = 14) had a “weak” rating, with few having a “moderate” (*n* = 4) or strong” (*n* = 1) rating. Meta-analyses were performed for the following outcomes: total physical activity, daily steps, moderate-to-vigorous physical activity (MVPA), fruit and vegetable consumption, sleep duration and sleep quality.

### Study characteristics

An overview of all study and participant characteristics is shown in Supplementary Table 2, and chatbot characteristics are shown in Supplementary Table 3. There was a total of 3567 participants and sample sizes ranged between 25 and 958. Mean participant age ranged between 9 and 71 (median = 44) years, and interventions ranged between 2 weeks and 1 year (median = 6 weeks). Samples consisted of apparently healthy adults (*n* = 8^24,31–37^), older adults (aged 65+, *n* = 1^38^), adolescents (*n* = 1^39^), families or parent-child dyads (*n* = 3^40–42^), physically inactive adults (*n* = 2^43,44^), adult cancer survivors (*n* = 1^45^), adults with a body mass index of 25–35 kg/m^2^ (*n* = 1^46^), adults with insomnia (*n* = 1^47^) and children with sleep difficulties (*n* = 1^48^). Retention (the proportion of enrolled participants who completed the intervention) ranged between 47.9% to 100% (median: 90.6%). Most interventions (*n* = 15) targeted physical activity, while 7 targeted healthy eating, 5 targeted sleep, and 3 targeted sedentary behaviour (of note, 8 studies targeted multiple behaviours). Over half of the interventions (*n* = 11) were chatbot only, while *n* = 8 were multi-component interventions. Multicomponent interventions were comprised of chatbots plus additional components such as pedometers or other wearable trackers (e.g., Fitbits), mailouts, access to intervention websites and diaries and logs. Eight of the chatbots were text-based outputs only, 3 were voice-based only, 8 involved combinations of text, voice, images, graphs and other visual displays such as an avatar (*n* = 1^33^), and the chatbots in 7^24,31,32,36,40,43,45^ studies involved AI or NLP. Six trials based their interventions on behaviour change models, which included the following: Social cognitive theory (SCT)^46^, the Player Experience and Need Satisfaction Model, the Agency, Challenge, Uncertainty, Discovery, and Outcomes framework and SCT^41^; the Capability, Opportunity, Motivation, Behaviour model^32,43^; SCT and the Transtheoretical Model^44^; and the Health Action Process Approach and Self-determination theory^34^. Behaviour change techniques most commonly integrated across the interventions included goal setting, self-monitoring, review of goals, problem-solving barriers, motivation, feedback and peer/social support (Supplementary Table 3).

### Meta-analyses results

**Fig. 2:**
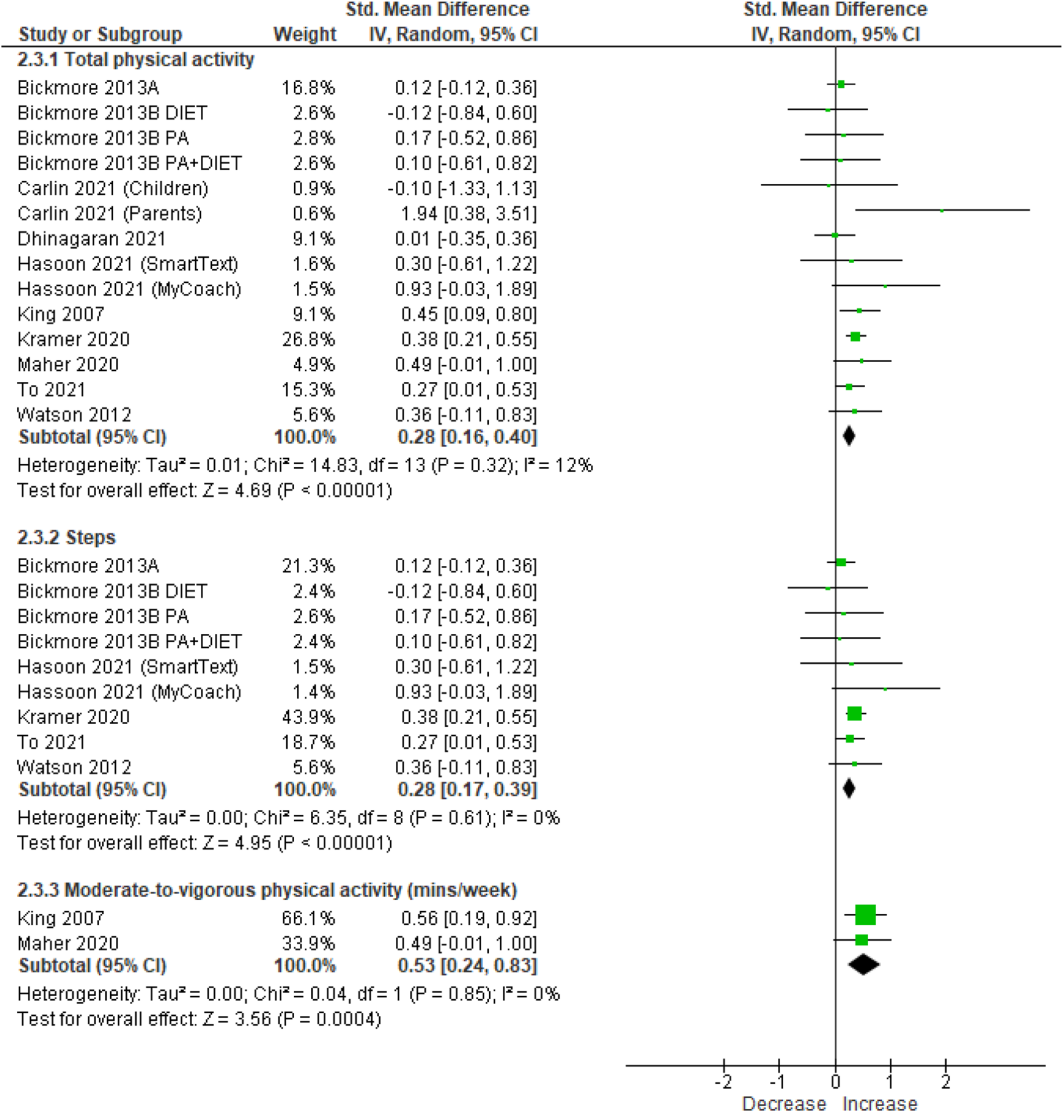
Meta-analysis results of the effect of chatbot interventions on total physical activity, steps and moderate-to-vigorous physical activity (using data from randomised controlled trials and single-group pre-post studies). Note: the meta-analysis of daily steps is a subset of total physical activity using Hedge’s g. 95% CI: 95% Confidence interval.

**Fig. 3:**
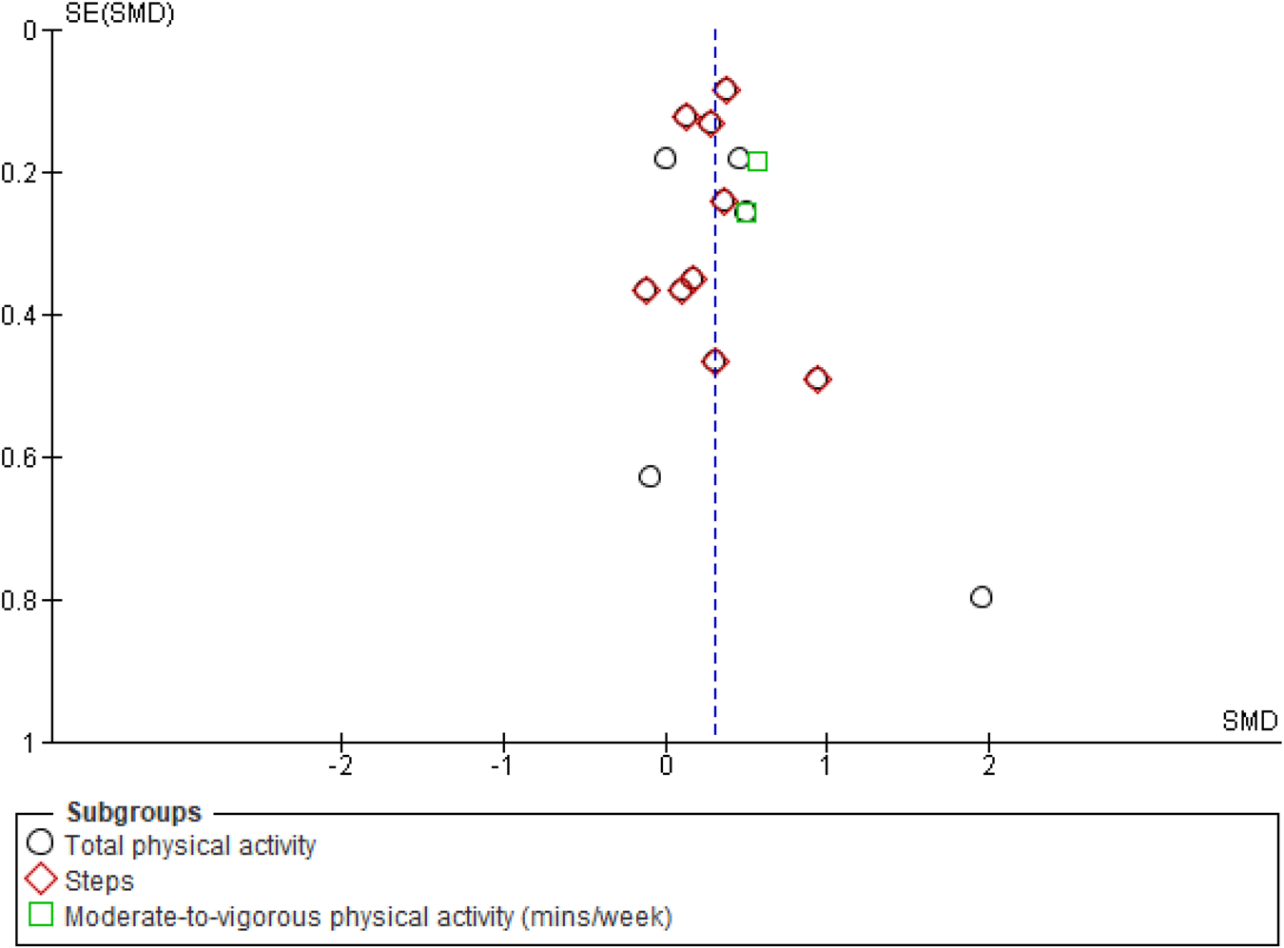
Funnel plot for studies that reported physical activity outcomes. SE (SMD): Standard error (standardized mean difference).

Meta-analyses of RCTs and pre-post trials showed significant effects of chatbot-based interventions for increasing total physical activity (SMD = 0.28 [95% CI = 0.16, 0.40], 10 studies, 1603 participants, I^2^ = 12%, *p* < 0.01 [Hedge’s g]), MVPA (SMD = 0.53 [95% CI = 0.24, 0.83], 2 studies, 184 participants, I^2^ = 0%, *p* < 0.01 [Hedge’s g]) and daily steps (SMD = 0.28 [95% CI = 0.17, 0.39], 6 studies, 1276 participants, I^2^ = 0%, *p* < 0·01 [Hedge’s g]) (Fig. 2). Meta-analysis using mean differences showed the increase in MVPA was 103 (95% CI = 48.20, 159.24) minutes per week and the increase in daily steps was 735 (95% CI = 441, 1029, *p* < 0.01 [Hedge’s g]) steps per day. Funnel plot evaluation of studies reporting physical activity outcomes suggested no evidence of publication bias (Fig. 3).

Meta-analyses of RCTs showed a significant and consistent effect in favour of chatbot interventions for total physical activity (SMD = 0.27 [95% CI = 0.07, 0.46], 6 studies, 641 participants, I^2^ = 14%, *p* < 0.01 [Hedge’s g]), and a small and consistent effect on daily steps which approached statistical significance (SMD = 0.18 [95% CI = 0.00, 0.36], 4 studies, 496 participants, I^2^ = 0%, *p* = 0.06 [Hedge’s g]) (Supplementary Fig. 1). Sensitivity analyses were undertaken for total physical activity, following the removal of an outlier. Results of sensitivity analyses showed the effect on total physical activity remained unchanged for meta-analyses of RCTs and pre-post studies (SMD = 0.28 [95% CI = 0.18, 0.38], *p* < 0.01 [Hedge’s g]) and RCTs only (SMD = 0.23 [95% CI = 0.07, 0.39], *p* < 0.01 [Hedge’s g]).

Overall, the grade of recommendation for chatbot interventions for increasing physical activity was Grade A: consistent level 1 studies.

**Fig. 4:**
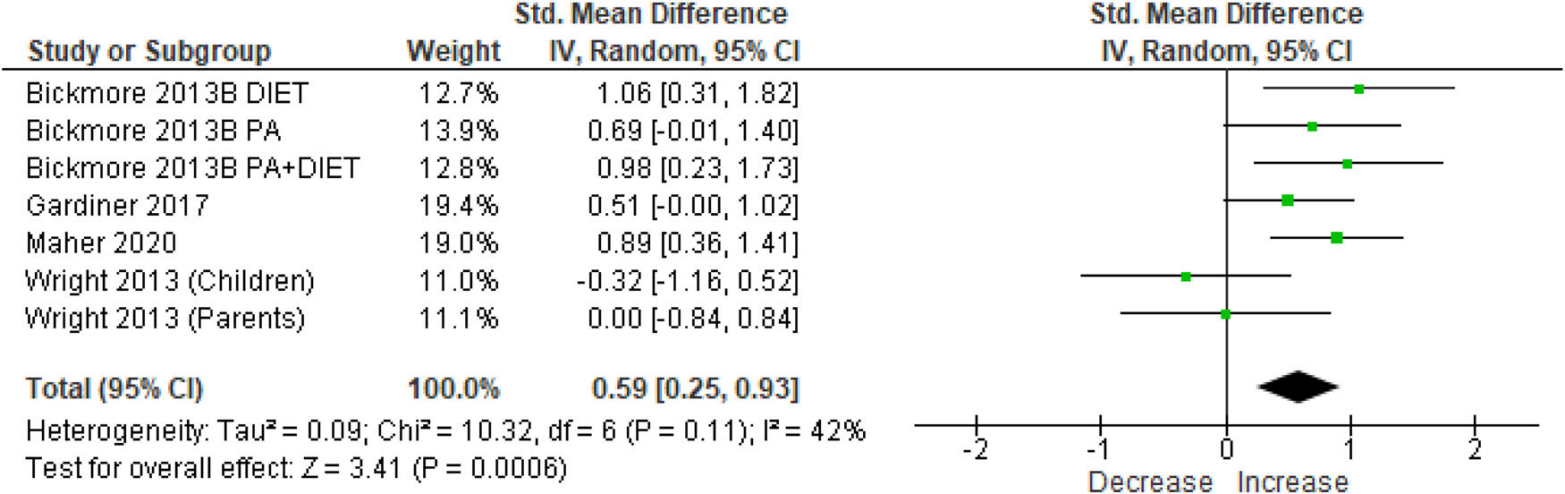
Forest plot of effect sizes of chatbots on fruit and vegetable consumption at post-intervention. Meta-analysis results of the effect of chatbot interventions on fruit and vegetable consumption (using data from randomised controlled trials and single-group pre-post studies) using Hedge’s g. 95% CI: 95% Confidence interval.

There was a significant effect of chatbot interventions for increasing fruit and vegetable consumption in meta-analyses of RCTs and pre-post studies (SMD = 0.59 [95% CI = 0.25, 0.93], 4 studies, 289 participants, I^2^ = 42%, *p* < 0.01, Fig. 4) and RCTs only (SMD = 0.52 [95% CI = 0.16, 0.87], 3 studies, 227 participants, I^2^ = 41%, *p* < 0.01 [Hedge’s g], Supplementary Fig. 1). Analyses using mean differences showed the effects equated to an increase in fruit and vegetable consumption by 1 serving per day (95% CI = 0.30, 1.68) for RCTs and pre-post studies, and 0.97 servings per day (95% CI = 0.15, 1.79) for RCTs only.

Overall, the grade of recommendation for chatbot interventions for increasing fruit and vegetable consumption is Grade A: consistent level 1 studies.

**Fig. 5:**
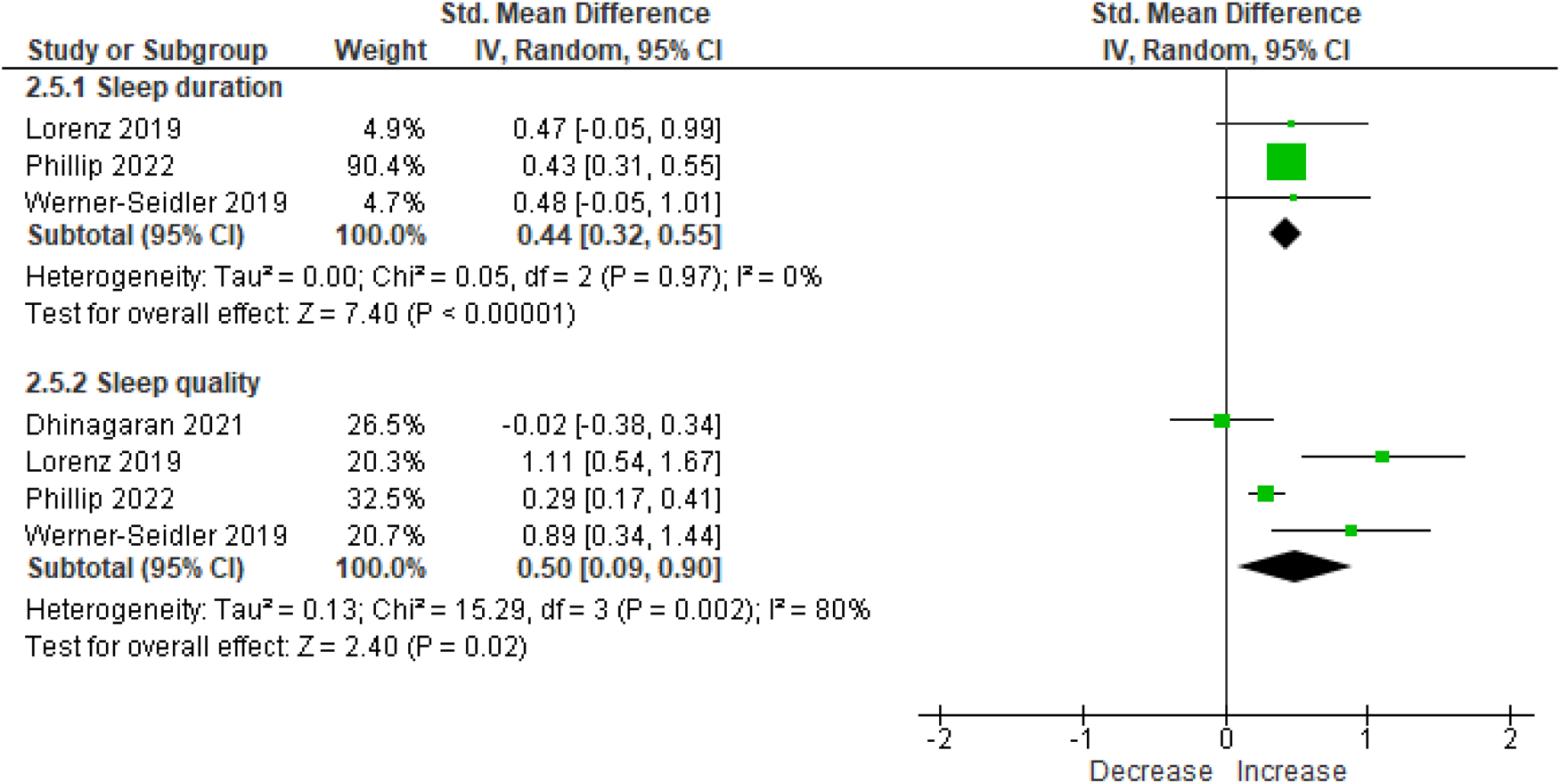
Forest plot of effect sizes of chatbots on sleep duration and sleep quality at post-intervention. Meta-analysis results of the effect of chatbot interventions on sleep duration and sleep quality (using data from randomised controlled trials and single-group pre-post studies) using Hedge’s g. 95% CI: 95% Confidence interval.

Significant increases were observed for sleep duration (SMD = 0.44 [95% CI = 0.32, 0.55], 3 studies, 1184 participants, I^2^ = 0%, *p* < 0.01 [Hedge’s g]) and sleep quality (SMD = 0.50 [95% CI = 0.09, 0.90], 4 studies, 1302 participants, I^2^ = 80%, *p* = 0.02, Fig. 5). Meta-analyses of sleep duration using mean differences showed the effect equated to an increase in sleep duration by 45 (95% CI = 33.2, 56.9) minutes per night.

The overall grade of recommendation for chatbot interventions for improving sleep duration and sleep quality is Grade B: consistent level 2 studies.

Results of subgroup analyses are shown in Supplementary Table 4. Subgroup analyses suggested that the effectiveness of chatbot interventions for increasing physical activity, daily steps or fruit and vegetable consumption did not differ based on intervention duration (≤6 weeks versus >6 weeks), intervention components (chatbot-only versus chatbot in a multicomponent intervention) and whether total physical activity was assessed using self-report or objective measures (all *p* > 0.05 [Hedge’s g]). Similarly, intervention effects on physical activity or sleep did not differ by chatbot output type (i.e., text versus speech/voice) or use of AI or NLP (all *p* > 0.05). However, a few significant subgroup effects were observed: multi-component interventions were more effective than chatbot-only interventions for improving sleep duration and sleep quality (*p* < 0.05 [Hedge’s g]); and text-based chatbots were more effective than speech/voice chatbots, and AI or NLP chatbots were more effective that non-AI or NLP chatbots for improving fruit and vegetable consumption (*p* < 0.05 [Hedge’s g]). There was an insufficient number of studies to perform analyses for the remaining subgroups.

## Discussion

This is the first systematic review and meta-analysis to comprehensively evaluate the effectiveness of chatbot interventions for improving physical activity, diet and sleep. We identified 19 trials involving 3567 participants, with findings suggesting that chatbot interventions are effective for increasing physical activity, fruit and vegetable consumption, sleep duration and sleep quality. The effects equated to increases of +735 steps per day, +1 serving of fruit and vegetables per day, and +45 min of sleep per night. Both short- and longer-term interventions and chatbot only and multicomponent interventions were effective. Further, there was evidence to suggest that text-based chatbots may be more effective than voice chatbots for improving fruit and vegetable consumption, and multicomponent interventions may be more effective for improving sleep duration and quality, compared with chatbot-only interventions.

Small-to-moderate effect size improvements (SMD range: 0.27 to 0.59) were observed for each of the health behaviours. These findings are comparable to previous meta-analyses, which have reported effect sizes (SMD) of between 0.13 to 0.68 for in-person, and various digital and web-based non-chatbot interventions for physical activity, diet (including fruit and vegetable consumption), and sleep^5–11,49–51^. All studies either based their intervention on a named behaviour change theory or model, or involved recognised behaviour change techniques which included goal setting, self-monitoring, review of goals, identifying and problem-solving barriers, motivation, feedback on goal attainment, peer/social support, and individualised advice and education. These behaviour change techniques have shown to be an important component of previous in-person and digital-based non-chatbot interventions designed for improving health behaviours^52–55^. The integration of these behaviour change techniques as part of the chatbot interventions may have contributed to improvements in the target behaviours, and helped overcome several barriers and challenges that have been previously identified in traditional in-person interventions such as limited interaction with a health professional, reduced motivation over time, and a lack of access to education and information.

Previous findings within healthcare settings indicates that chatbots offer low intensity information delivery services and can deliver behaviour change interventions into existing, daily clinical settings, while minimising additional burden on existing healthcare providers^27,56^. Various platforms were used to deliver the chatbots in our present review, including standalone websites, study specific smartphone apps, or they were deployed via third party integration with instant messaging apps (e.g., Messenger [Meta Platforms, Inc; Menlo Park, CA] and Slack [Slack Technologies; San Francisco, CA]) or smart speakers (e.g., Amazon Echo smart speakers). Therefore, chatbots have great potential to be integrated within many commonly used platforms and accessed at any time by users, and place little (if any) strain on existing resources such as staff, time, money, and workload. However, potential drawbacks may include limits to functionality (e.g., the chatbot is unable to respond to the user’s question) and the inability to continue using the chatbot when the communication platforms are updated. Furthermore, it is also important to consider that digital determinants of health highlight how digital technologies and online platforms can impact health outcomes and behaviours^57^. Technology use has become more common in recent times, including among population groups that previously had lower access (e.g., older adults). Likewise, digital technologies provide the ability to reach specific populations that would be extremely hard to reach otherwise (e.g., people with rare diseases that are geographically dispersed). While digital health tools have the potential to improve health outcomes, reduce disparities, and increase access to healthcare services, not all populations have equal access to these tools. This can further exacerbate existing health disparities, particularly for marginalized individuals who may lack access to technology or digital literacy. A potential concern is that digital health tools such as chatbots may exclude those who are already marginalized or have limited access to technology, such as individuals experiencing homelessness or elderly individuals with limited digital literacy. To ensure that digital health tools such as chatbots are accessible and beneficial for everyone, it is crucial to include diverse populations in future studies.

The chatbot interventions included in our review were text and voice-based, and many included a variety of visual or graphical images and displays (e.g., graphs of weekly physical activity) and avatars. Approximately one-third of the interventions evaluated allowed user-initiated free flow conversations with the chatbot, and most of the chatbots also sent daily reminders, prompts, goals and/or informational messages. Our subgroup analyses showed no difference in text-based or speech/voice-based chatbots for improving physical activity or sleep, however text-based chatbots were more effective than voice-based chatbots for improving fruit and vegetable consumption. This may be because many of the text-based chatbots included in our review were implemented through smartphones, which can be used anytime throughout the day (e.g., at home, whilst shopping for groceries, while commuting or during work), while the voice-based chatbots were typically limited to home-use (i.e., Amazon Echo smart speakers^40,45^). However, previous work has identified that voice interaction allows improved engagement with chatbots, through a more convenient hands-free interaction^58,59^, and therefore, this represents an area for future research. Previous work has also highlighted the need for chatbots to establish appropriate rapport or relationships with users through individualised and compassionate interactions for a sustained and engaging intervention^20^. This can enhance the chatbots’ ability to provide a personalised and human-like interaction experience, and can improve user experience through the delivery of individualised interventions and educational content and information^20^.

Findings from this systematic review and meta-analysis highlight the potential for chatbots to be implemented across diverse populations in different settings. There were a range of populations, including individuals with cancer, insomnia, and individuals who were sedentary, overweight or obese, ranging in mean age between 9 and 72 years. The interventions ranged between 2 weeks and 1 year and retention was generally high (median: 90.6%, range: 47.9% to 100%). However, most of the included studies (58%) were 6 weeks or less and previous research has suggested adherence to wearable devices for monitoring lifestyle behaviours decreases up to two years following a short-term intervention^60^. Previous work has identified that a key strength of chatbots is the ability to replicate interaction with a human or health professional, to provide a safe environment for the users to discuss, share, and ask for information on sensitive issues^24,61,62^. AI algorithms are now becoming more able to perceive an understanding of human emotions which can make it easier for users to share sensitive information^63^. Therefore, chatbots are strong candidates for implementation across large and diverse samples for lifestyle education, promotion of health behaviour change, and can be a tool for clinicians to use to offer healthcare services to prevent and manage various health conditions at the population level, and as an intervention for clinical or vulnerable populations. However, it is important to consider that despite the fact that AI algorithms are improving in their ability to respond to human interaction, they cannot truly empathize and are limited in their understanding of a user’s complete situation. Consequently, users may reveal sensitive information to an AI algorithm that lacks the capacity to handle such information suitably. Privacy issues can arise when using chatbots. Chatbots may collect and store personal and even intimate data in the form of chat logs, user preferences, and behavioral patterns. AI-based chatbots will often retain user interaction data as training material. Users may sometimes share sensitive information with chatbots, assuming they are interacting with a human. Unintended data exposure can occur through hacking, sharing with third parties, or lax security. Chatbots should have clear and transparent privacy policies, preferably leaving decisions around data retention in the hands of the user. Furthermore, as AI algorithms become more adept at mimicking empathy in chatbots, it is essential to ensure that appropriate security measures and regulations are put in place to safeguard against potential privacy violations from unauthorized parties gaining access to sensitive information.

Recent findings have shown that current deidentification practices for accelerometer-measured physical activity data are insufficient to ensure privacy^64^ and health data breaches have increased over the past decade^65^. Masking individual data points by grouping data points with similar characteristics is no longer a reliable approach in modern data landscapes where data is easily linked across multiple sources. Additionally, any data that can be combined with other information is considered personal identifiable information, making it difficult to distinguish between data categories. To safeguard personal records against revealing individual identities, more advanced techniques are necessary beyond simply categorizing data as personal identifiable information or not. These findings have important implications for the use of chatbots for improving lifestyle behaviours, highlighting the need for robust data privacy measures to protect user privacy. Furthermore, there are potential privacy concerns with emerging technologies like chatbots offered to patients due to the discrepancy between standard medical care practices and technology’s terms of use^66^. Patients may not fully understand the implications of sharing personal information with chatbots, which may collect data beyond their expectations and control. It is also important to consider that vendors may not provide enough information to consumers about data privacy risks, while healthcare providers are aware of the issue but face challenges in properly managing the risk-^67,68^. Providers struggle to contract the risk properly, which may result in potential breaches of patient privacy.

Evidence to-date suggests that chatbots may be a powerful tool for helping people make positive health behaviour changes, however the field of research is in its infancy. The following recommendations for future research may help guide health researchers and human-computer interaction researchers as they design, evaluate and implement chatbots for behaviour change:

1. Studies with large sample sizes are needed, to ensure that studies are sufficiently powered to detect improvements (particularly in downstream physiological and psychological benefits that may flow from health behaviour change and are typically smaller in magnitude).
2. High-quality research designs, such as RCTs, are needed to definitively evaluate effectiveness. Variants of traditional RCTs, such as micro RCTs, may allow the effects of multi-component interventions to be disentangled.
3. Such studies should employ high-quality outcome measures (such as device-measured physical activity) to reduce the potential for measurement bias.
4. Longer-term follow-up is needed (i.e., to evaluate effects to 12 months and beyond).
5. Ongoing work is needed to evaluate different aspects of the chatbot user-experience (such as tone, personality, text-based vs. voice-based, and frequency of communication).
6. In a similar vein, ongoing work is needed to evaluate chatbots for health behaviour change as their capabilities expand, due to advances in the fields of conversational AI and machine learning. Such advances will allow chatbots to learn from interactions and improve their responses over time, and generate their own responses based on the context of the conversation and the users’ input, which are likely to lead to improved accuracy, personalisation and efficacy.
7. Health behaviour chatbot programs should be designed with longer-term implementation in mind (e.g., research/tech industry/health care system partnerships). This will help ensure that intervention components are scalable and will help ensure a pipeline of new users (e.g., if the intervention is embedded into health care services).

A key strength of this work is its innovation, as the first systematic review and meta-analysis to evaluate the effectiveness of chatbots for improving physical activity, diet and sleep. The highest quality systematic review methods were used, including searching 14 different databases; completing screening, data extraction and study quality assessment in duplicate; the use of meta-analytic synthesis; meta-analytic sensitivity analyses; and completion of additional steps added in PRISMA 2020 — certainty of evidence and assessment of reporting bias. The study’s conclusions are limited by the current evidence base. Over half of the studies (*n* = 11, 58%) had a sample size of 75 or less and there was limited evidence examining longer-term impacts of chatbots, with most studies (*n* = 11, 58%) being 6 weeks or less, and no interventions being longer than 1 year. It is also important to recognise that a lack of power in our subgroup analyses may have prevented us from identifying associations that are present, but not identified in our findings. Furthermore, most of the studies involved recruiting participants from the general public (via self-referral) and it is possible that individuals who expressed interest in participating in the trials were more likely to be technologically-savvy and therefore our findings may be limited in generalisability to those with lower levels of digital literacy.

Findings from this systematic review and meta-analysis indicate that chatbot interventions are effective for increasing physical activity, fruit and vegetable consumption, sleep duration and sleep quality. Chatbot interventions were effective across a range of populations and age groups, with shorter- and longer-term, text and voice-based chatbots, chatbot-only and multicomponent interventions being effective. However, future large-scale trials, with rigorous study designs and outcome measures, and long-term follow-up are required to confirm these findings.

## Methods

### Protocol and registration

The protocol for this systematic review was prospectively registered on PROSPERO (ID: CRD42022353633) and the results are reported according to PRISMA^69^ guidelines. There was no funding source for this study.

### Selection criteria and search strategy

The eligibility criteria were developed using the population, intervention, comparison, outcomes and study type (PICOS)^70^ framework as follows: Population: any population; Intervention: Any intervention targeting physical activity, sedentary behaviour, diet or sleep, that involved a chatbot. The following definition of chatbot was used: Chatbots, also known as conversational agents, employ dialog systems to enable natural language conversations with users using speech and/or text^21^. This included any type of text and/or voice/speech-based chatbot (with or without visual output, e.g., graphs) operating as standalone software or via a web browser or mobile application, and/or diverse platforms (e.g., Slack, Facebook messenger, WhatsApp, virtual/embodied agent, SMS). Studies were eligible irrespective of intervention modality (i.e., standalone software or web-browser), supervision or setting (i.e., used in a laboratory setting vs. at home vs. healthcare setting) or dose (frequency, intensity and duration). Comparator: Studies were eligible if they involved any comparison condition (e.g., usual care or an equal attention intervention), or no comparison (e.g., single group pre-post studies). Outcomes: Any outcome related to physical activity, diet or sleep. Study type: Any experimental study design.

Fourteen electronic databases were searched (ACM Digital Library, CINAHL, The Cochrane Library, Embase, Emcare, IEEE Xplore, JMIR publications, MEDLINE, Ovid, ProQuest central, ProQuest Nursing and Allied Health Source, PsycINFO, PubMed and Scopus) using subject heading, keyword and MeSH term searches for “chatbot”, “physical activity”, “sedentary behaviour”, “sleep”, and “diet” (see Supplementary table 5 for the full search strategy). Database searches were limited to peer-reviewed journal articles published in English-language from inception until 1st September, 2022.

### Data management and extraction

Search results were imported into EndNote x9 (Clarivate, Philadelphia, PA), duplicates were removed, and articles where then exported into Covidence (Veritas Health Innovation, Melbourne, Australia). All stages of screening were completed independently, and in duplicate by 10 authors (AW, AM, BS, CS, DD, EE, JB, KS, LM, RV), with disagreements resolved by discussion with the senior author (CM). Data extraction was undertaken in duplicate by 10 authors independently (AW, AM, BS, CS, DD, EE, JB, KS, LM, RV), with disagreements resolved by discussion between authors. Standardised data extraction forms were used to extract the following information from eligible studies: study characteristics (e.g., study design, sample size), intervention details (type of chatbot and intervention dose), outcome measures, overall results for relevant outcomes. Study quality and risk of bias was assessed by independent reviewers in duplicate using the Effective Public Health Practice Project Quality Assessment (EPHPP) tool^71^, with discrepancies resolved through discussion between authors. The EPHPP evaluates six aspects of design and methods, which include selection bias, study design, confounders, blinding, data collection methods, and withdrawals and dropouts. These factors contribute to the overall rating given by the tool. Each of these dimensions is assessed using a three-point scale, with ratings of strong, moderate, or weak. In addition to these six dimensions, the EPHPP also considers intervention integrity and analyses, although these are not included in the calculation of the global rating. Using the EPHPP tool, studies were rated as weak, moderate, or strong in the components of (1) selection bias, (2) study design, (3) confounders, (4) blinding, (5) data collection, and (6) withdrawals.

### Outcomes of interest

Outcomes of interest were (1) total physical activity (any measure of low, moderate and/or vigorous intensity physical activity reported as a duration, e.g., minutes per day or week), (2) moderate-to-vigorous physical activity only (MVPA, minutes/week), (3) daily steps, (4) fruit and vegetable consumption, (5) sleep quality and (6) sleep duration.

### Meta-analysis methods

For each meta-analysis, data were combined at the study level. Separate meta-analyses were performed using data from: (i) all studies (RCTs and single-group pre-post studies) and (ii) RCTs only. Outcomes of interest were analysed as continuous variables and data were pooled using: (1) pre- and immediate post-intervention means and standard deviations (SDs) (for single group pre-post studies and RCTs) and (2) immediate post-intervention means and SDs for the intervention and usual care groups (for the RCT only analysis). There was insufficient data to conduct meta-analyses of follow-up timepoints. Standardised mean differences (SMDs) were used as the effect measure for meta-analyses, to allow comparison of data from different scales. If means and SDs were not reported in a study, authors were contacted or means and SD were calculated based on available data, using recommended formulas (e.g., using sample size, median and range)^72^. If multiple methods of assessing an outcome were used in a study, the method that was either the gold standard, or the method with established reliability and validity was used for the meta-analysis. All meta-analyses were performed using RevMan software (version 5).

Publication bias was evaluated using funnel plots of SMDs and standard errors and evaluating for asymmetries or missing sections within the plot, for meta-analyses that involved more than 10 studies^73^. The Cochran’s Q test was used to assess statistical heterogeneity and the I^2^ statistic to quantify the proportion of the overall outcome attributed to variability^73^. The following cut-off values for the I^2^ statistic were used: 0 to 29% = no heterogeneity; 30 to 49% = moderate heterogeneity; 50 to 74% = substantial heterogeneity; and 75 to 100% = considerable heterogeneity^73^. The following subgroup analyses were undertaken: (1) intervention components (chatbot only versus multi-component interventions), (2) output type (speech/voice versus text), (3) use of AI or NLP (yes versus no), and (4) duration (6 weeks or less versus more than 6 weeks). Standardised classifications for the magnitude of effect were used (0.20 = small effect; 0.20 to 0.50 = medium effect; and greater than 0.50 = large effect)^74^. A *p*-value of <0.05 was considered statistically significant.

The overall level of evidence was graded using the Oxford Centre for Evidence-Based Medicine 2011 Levels of Evidence^75^, as follows: grade A: consistent level 1 studies (i.e., individual RCTs); B: consistent level 2 (i.e., individual cohort studies) or 3 studies (i.e., individual case-control studies) or extrapolations from level 1 studies; C: level 4 studies (i.e., case series) or extrapolations from level 2 or 3 studies; or D: level 5 (i.e., expert opinion without explicit critical appraisal) evidence or inconsistent or inconclusive studies of any level^70^.

### Deviations from the registered protocol

We planned to include sedentary behaviour as an outcome of interest, however there was insufficient data in the included studies to perform a meta-analysis on this outcome. Furthermore, following registration of our protocol, we decided to add subgroup analyses for output type and AI or NLP use, as it would be a valuable addition, and may help guide future research on chatbot development.

### Reporting summary

Further information on research design is available in the Nature Research Reporting Summary linked to this article.

## Supplementary information

Supplementary material

Reporting Summary

## Data Availability

BS has full access to all of the data in the study and takes responsibility for the integrity of the data and the accuracy of the data analysis. All study materials are available from the corresponding author upon reasonable request.

## References

[1] Kohl H (2012). The pandemic of physical inactivity: global action for public health. Lancet.

[2] Stamatakis E (2019). Is the time right for quantitative public health guidelines on sitting? A narrative review of sedentary behaviour research paradigms and findings. Br. J. Sports Med..

[3] Endalifer ML, Diress G (2020). Epidemiology, predisposing factors, biomarkers, and prevention mechanism of obesity: a systematic review. J. Obes..

[4] Bloom, D. et al. The global economic burden of noncommunicable diseases: program on the global demography of aging. PGDA Working Papers 8712, Program on the Global Demography of Aging (2012).

[5] Conn V, Hafdahl A, Mehr D (2011). Interventions to increase physical activity among healthy adults: meta-analysis of outcomes. Am. J. Public Health.

[6] Kang M, Marshall SJ, Barreira T, Lee J (2009). Effect of pedometer-based physical activity interventions: a meta-analysis. Res Q Exerc Sport..

[7] Chase J (2015). Interventions to increase physical activity among older adults: a meta-analysis. Gerontologist.

[8] Bull E, Dombrowski S, McCleary N, Johnston M (2014). Are interventions for low-income groups effective in changing healthy eating, physical activity and smoking behaviours? A systematic review and meta-analysis. BMJ Open..

[9] Delgado-Noguera M, Tort S, Martínez-Zapata M, Bonfill X (2011). Primary school interventions to promote fruit and vegetable consumption: A systematic review and meta-analysis. Prev. Med..

[10] Murawski B, Wade L, Plotnikoff R, Lubans D, Duncan M (2018). A systematic review and meta-analysis of cognitive and behavioral interventions to improve sleep health in adults without sleep disorders. Sleep. Med. Rev..

[11] Blake M, Sheeber L, Youssef G, Raniti M, Allen N (2017). Systematic review and meta-analysis of adolescent cognitive–behavioral sleep interventions. Clin. Child Fam. Psychol. Rev..

[12] Sadeghi-Bazargani H, Tabrizi JS, Azami-Aghdash S (2014). Barriers to evidence-based medicine: a systematic review. J. Evaluation Clin. Pract..

[13] Kelly S (2016). Barriers and facilitators to the uptake and maintenance of healthy behaviours by people at mid-life: a rapid systematic review. PLoS One.

[14] Müller AM, Alley S, Schoeppe S, Vandelanotte C (2016). The effectiveness of e-& mHealth interventions to promote physical activity and healthy diets in developing countries: a systematic review. Int. J. Behav. Nutr. Phys. Act..

[15] Arroyo AC, Zawadzki MJ (2022). The implementation of behavior change techniques in mhealth apps for sleep: systematic review. JMIR Mhealth Uhealth..

[16] Fiedler J, Eckert T, Wunsch K, Woll A (2020). Key facets to build up eHealth and mHealth interventions to enhance physical activity, sedentary behavior and nutrition in healthy subjects – an umbrella review. BMC Public Health.

[17] Michie S, Yardley L, West R, Patrick K, Greaves F (2017). Developing and evaluating digital interventions to promote behavior change in health and health care: recommendations resulting from an international workshop. J. Med Internet Res..

[18] Grady A (2018). Improving the public health impact of eHealth and mHealth interventions. Aust. N. Z. J. Public Health.

[19] McCarroll R, Eyles H, Ni Mhurchu C (2017). Effectiveness of mobile health (mHealth) interventions for promoting healthy eating in adults: A systematic review. Preventive Med..

[20] Aggarwal A, Tam CC, Wu D, Li X, Qiao S (2023). Artificial Intelligence-Based Chatbots for Promoting Health Behavioral Changes: Systematic Review. J. Med. Internet Res..

[21] Laranjo L (2018). Conversational agents in healthcare: a systematic review. J. Am. Med Inf. Assoc..

[22] Milne-Ives M (2018). The effectiveness of artificial intelligence conversational agents in health care: systematic review. J. Med Internet Res..

[23] Davenport T, Kalakota R (2019). The potential for artificial intelligence in healthcare. Future Health. J..

[24] Maher C, Davis C, Curtis R, Short C, Murphy K (2020). A physical activity and diet program delivered by artificially intelligent virtual health coach: proof-of-concept study. JMIR Mhealth Uhealth..

[25] Abd-Alrazaq A, Rababeh A, Alajlani M, Bewick B, Househ M (2020). Effectiveness and safety of using chatbots to improve mental health: systematic review and meta-analysis. J. Med Internet Res..

[26] Vaidyam A, Wisniewski H, Halamka J, Kashavan M, Torous J (2019). Chatbots and conversational agents in mental health: a review of the psychiatric landscape. Can. J. Psychiatry.

[27] Provoost S, Lau H, Ruwaard J, Riper H (2017). Embodied conversational agents in clinical psychology: a scoping review. J. Med Internet Res..

[28] Sezgin E, Militello L, Huang Y, Lin S (2020). A scoping review of patient-facing, behavioral health interventions with voice assistant technology targeting self-management and healthy lifestyle behaviors. Transl. Behav. Med..

[29] Luo TC, Aguilera A, Lyles CR, Figueroa CA (2021). Promoting physical activity through conversational agents: mixed methods systematic review. J. Med. Internet Res..

[30] Oh Y, Zhang J, Fang M, Fukuoka Y (2021). A systematic review of artificial intelligence chatbots for promoting physical activity, healthy diet, and weight loss. Int. J. Behav. Nutr. Phys. Act..

[31] Bickmore T, Schulman D, Sidner C (2013). Automated interventions for multiple health behaviors using conversational agents. Patient Educ. Couns..

[32] Dhinagaran D (2021). Conversational agent for healthy lifestyle behavior change: web-based feasibility study. JMIR Form. Res..

[33] Friederichs S, Bolman C, Oenema A, Guyaux J, Lechner L (2014). Motivational interviewing in a Web-based physical activity intervention with an avatar: randomized controlled trial. J. Med. Internet Res..

[34] Kramer J (2020). Which components of a smartphone walking app help users to reach personalized step goals? Results from an optimization trial. Ann. Behav. Med..

[35] Philip P (2020). Efficacy of a smartphone-based virtual companion to treat insomniac complaints in the general population: sleep diary monitoring versus an internet autonomous intervention. J. Clin. Med..

[36] Carfora V, Bertolotti M, Catellani P (2019). Informational and emotional daily messages to reduce red and processed meat consumption. Appetite.

[37] Gardiner P (2017). Engaging women with an embodied conversational agent to deliver mindfulness and lifestyle recommendations: a feasibility randomized control trial. Patient Educ. Couns..

[38] Bickmore T (2013). A randomized controlled trial of an automated exercise coach for older adults. J. Am. Geriatr. Soc..

[39] Cushing C (2021). Adaptive mHealth intervention for adolescent physical activity promotion. J. Pediatr. Psychol..

[40] Carlin A, Logue C, Flynn J, Murphy M, Gallagher A (2021). Development and feasibility of a family-based health behavior intervention using intelligent personal assistants: randomized controlled trial. JMIR Form. Res..

[41] De-Jongh González O (2022). The Aim2Be mHealth intervention for children with overweight or obesity and their parents: person-centered analyses to uncover digital phenotypes. J. Med. Internet Res..

[42] Wright J (2013). Randomized trial of a family-based, automated, conversational obesity treatment program for underserved populations. Obesity.

[43] To Q, Green C, Vandelanotte C (2021). y. JMIR Mhealth Uhealth..

[44] King A (2021). Ongoing physical activity advice by humans versus computers: the community health advice by telephone (CHAT) trial. Health Psychol..

[45] Hassoon A (2021). Randomized trial of two artificial intelligence coaching interventions to increase physical activity in cancer survivors. NPJ Digit. Med..

[46] Watson A, Bickmore T, Cange A, Kulshreshtha A, Kvedar J (2012). An internet-based virtual coach to promote physical activity adherence in overweight adults: randomized controlled trial. J. Med. Internet Res..

[47] Lorenz N, Heim E, Roetger A, Birrer E, Maercker A (2019). Randomized controlled trial to test the efficacy of an unguided online intervention with automated feedback for the treatment of insomnia. Behav. Cogn. Psychother..

[48] Werner-Seidler A (2019). Pilot evaluation of the Sleep Ninja: a smartphone application for adolescent insomnia symptoms. BMJ Open..

[49] Robert C (2021). Effectiveness of eHealth nutritional interventions for middle-aged and older adults: systematic review and meta-analysis. J. Med. Internet Res..

[50] Champion K (2019). Effectiveness of school-based eHealth interventions to prevent multiple lifestyle risk behaviours among adolescents: a systematic review and meta-analysis. Lancet Digital Health.

[51] Direito A, Carraça E, Rawstorn J, Whittaker R, Maddison R (2017). mHealth Technologies to influence physical activity and sedentary behaviors: behavior change techniques, systematic review and meta-analysis of randomized controlled trials. Ann. Behav. Med..

[52] Samdal G, Eide G, Barth T, Williams G, Meland E (2017). Effective behaviour change techniques for physical activity and healthy eating in overweight and obese adults; systematic review and meta-regression analyses. Int. J. Behav. Nutr. Phys. Act..

[53] Schroé H (2020). Which behaviour change techniques are effective to promote physical activity and reduce sedentary behaviour in adults: a factorial randomized trial of an e- and m-health intervention. Int. J. Behav. Nutr. Phys. Act..

[54] Awoke M (2022). Behaviour change techniques in weight gain prevention interventions in adults of reproductive age: meta-analysis and meta-regression. Nutrients.

[55] Noordman J, van der Weijden T, van Dulmen S (2012). Communication-related behavior change techniques used in face-to-face lifestyle interventions in primary care: A systematic review of the literature. Patient Educ. Couns..

[56] Xu L, Sanders L, Li K, Chow J (2021). Chatbot for health care and oncology applications using artificial intelligence and machine learning: systematic review. JMIR Cancer.

[57] van Kessel R, Wong BLH, Clemens T, Brand H (2022). Digital health literacy as a super determinant of health: more than simply the sum of its parts. Internet Interv..

[58] Chew H (2022). The use of artificial intelligence-based conversational agents (chatbots) for weight loss: scoping review and practical recommendations. JMIR Med. Inform..

[59] Stephens TN, Joerin A, Rauws M, Werk LN (2019). Feasibility of pediatric obesity and prediabetes treatment support through Tess, the AI behavioral coaching chatbot. Transl. Behav. Med..

[60] Hartman SJ (2022). Fitbit use and activity levels from intervention to 2 years after: secondary analysis of a randomized controlled trial. JMIR Mhealth Uhealth..

[61] Galvão Gomes da Silva J (2018). Experiences of a motivational interview delivered by a robot: qualitative study. J. Med Internet Res..

[62] Carrasco-Hernandez L (2020). A mobile health solution complementing psychopharmacology-supported smoking cessation: randomized controlled trial. JMIR Mhealth Uhealth..

[63] Shin D (2021). The perception of humanness in conversational journalism: an algorithmic information-processing perspective. N. Media Soc..

[64] Na L (2018). Feasibility of Reidentifying Individuals in Large National Physical Activity Data Sets From Which Protected Health Information Has Been Removed With Use of Machine Learning. JAMA Netw Open.

[65] McCoy TH, Perlis RH (2018). Temporal Trends and Characteristics of Reportable Health Data Breaches, 2010-2017. JAMA.

[66] Rosenfeld L, Torous J, Vahia IV (2017). Data Security and Privacy in Apps for Dementia: An Analysis of Existing Privacy Policies. Am. J. Geriatr. Psychiatry.

[67] Koppel, R. & Kreda, D. Health care information technology vendors’ “hold harmless” clause: implications for patients and clinicians. *JAMA* **301**, 1276–1278 (2009). 10.1001/jama.2009.398 19318655

[68] Goodman, K. W. et al. AMIA Board of Directors. Challenges in ethics, safety, best practices, and oversight regarding HIT vendors, their customers, and patients: a report of an AMIA special task force. *J. Am. Med. Inform. Assoc.* **18**, 77–81 (2011). 10.1136/jamia.2010.008946 PMC3005880 21075789

[69] Page M (2021). The PRISMA 2020 statement: an updated guideline for reporting systematic reviews. BMJ.

[70] Schardt C, Adams M, Owens T, Keitz S, Fontelo P (2007). Utilization of the PICO framework to improve searching PubMed for clinical questions. BMC Med Inf. Decis. Mak..

[71] Thomas BH, Ciliska D, Dobbins M, Micucci S (2004). A process for systematically reviewing the literature: providing the research evidence for public health nursing interventions. Worldviews Evid. Based Nurs..

[72] Hozo S, Djulbegovic B, Hozo I (2005). Estimating the mean and variance from the median, range, and the size of a sample. BMC Med. Res. Methodol..

[73] Higgins, J. et al. (editors). Cochrane Handbook for Systematic Reviews of Interventions version 6.3 (updated February 2022). (2022) www.training.cochrane.org/handbook.

[74] Cohen, J. Statistical Power Analysis for the Behavioral Sciences (2nd ed.). (1988).

[75] Howick, J. et al. Oxford centre for evidence-based medicine levels of evidence. (2011) https://www.cebm.ox.ac.uk/resources/levels-of-evidence/ocebm-levels-of-evidence.

