# Supplementary material for: Systematic review and meta-analysis of the effectiveness of chatbots on lifestyle behaviours
Source: NPJ Digit Med. 2023 Jun 23;6:118. doi: 10.1038/s41746-023-00856-1 (PMC10290125; doi:10.1038/s41746-023-00856-1)
Supplement: Supplementary file 1 — Supplementary material [file 41746_2023_856_MOESM1_ESM.docx]

# Supplementary Table 1. Search strategy

| Search category | Search terms |
| --- | --- |
| Virtual assistant | “chatbot*” OR “chat bot” OR “chat-bot” OR “chatter bot” OR “chatterbot” OR “chatter robot” OR “artificial intelligence” OR “virtual agent” OR “bot” OR “conversational bot*” OR “conversational agent*” OR “conversational system*” OR “conversational interface” OR “conversational AI” OR “conversational agency” OR “dialog system*” OR “dialogue system*” OR “assistance technolog*” OR “relational agent*” OR “virtual agent*” OR “virtual assistant*” OR “virtual coach” |
| Health behaviours | Physical activity:  “physical activit*” OR “exercise” OR “exercis*” OR “sport*” OR “MVPA” OR “walk*” OR “health behav*” OR “behavior change” OR behaviour change” OR “physical training” OR “outdoor activity” OR “fitness” OR “strength exercise” OR “resistance training” OR “weight-lifting” OR “weight lifting” OR “bicycle*” OR “cycl*” OR “swim*” OR “run*” or “jog*” OR “play*” OR “lifestyle”  Sedentary behaviour:  “sedentary lifestyle” OR “sedentar*” OR “sitting” OR “screen time” OR “inactiv*” OR “television” OR “TV” OR “Video games” OR “video gam*” OR “health promotion” OR “health behav*” OR “behav* change”  Sleep:  “sleep” OR “insomnia”  Diet/nutrition:  “diet” OR “nutrition*” OR “healthy eating” OR “food habits” OR “fruit” OR “vegetable” OR “snack food*” OR “snack*” OR “soft drink*” OR “carbonated beverages” OR “discretionary food*” OR “junk food*” OR “eat*” OR “weight*” OR “overweight” OR “over-weight” OR “obesity” OR “bodyweight” OR “body composition*” OR “BMI” OR “body mass index” |

# Supplementary Table 2. Study quality ratings and risk of bias assessed using the Effective Public Heath Practice Project Quality Assessment Tool.

| Study | Selection Bias | Study Design | Confounders | Blinding | Data Collection Methods | Withdrawals and Dropouts | Overall Rating* |
| --- | --- | --- | --- | --- | --- | --- | --- |
| Bickmore 2013A | Moderate | Strong | Strong | Moderate | Strong | Strong | Strong |
| Bickmore 2013B | Weak | Strong | Strong | Weak | Strong | Strong | Weak |
| Carlin 2021 | Weak | Strong | Weak | Weak | Strong | Moderate | Weak |
| Carfora 2019 | Weak | Strong | Strong | Weak | Strong | Strong | Weak |
| Cushing 2021 | Weak | Strong | Strong | Weak | Strong | Strong | Weak |
| De-Jongh González 2022 | Moderate | Strong | Weak | Weak | Strong | Strong | Weak |
| Dhinagaran 2021 | Weak | Moderate | Strong | Weak | Strong | Strong | Weak |
| Friederichs 2014 | Weak | Strong | Weak | Weak | Strong | Weak | Weak |
| Gardiner 2017 | Weak | Strong | Strong | Weak | Strong | Strong | Weak |
| Hassoon 2021 | Moderate | Strong | Strong | Weak | Strong | Strong | Moderate |
| King 2007 | Weak | Strong | Strong | Weak | Strong | Strong | Weak |
| Kramer 2020 | Weak | Moderate | Strong | Weak | Strong | Strong | Weak |
| Lorenz 2019 | Weak | Strong | Strong | Weak | Strong | Strong | Weak |
| Maher 2020 | Moderate | Moderate | Strong | Weak | Strong | Strong | Moderate |
| Phillip 2022 | Weak | Strong | Strong | Weak | Strong | Weak | Weak |
| To 2021 | Moderate | Moderate | Strong | Weak | Strong | Moderate | Moderate |
| Watson 2012 | Weak | Strong | Strong | Weak | Strong | Strong | Weak |
| Werner-Seidler 2019 | Weak | Moderate | Strong | Weak | Strong | Moderate | Weak |
| Wright 2013 | Moderate | Strong | Strong | Weak | Strong | Strong | Moderate |
| ∗Strong overall rating = 0 weak sub-ratings; moderate overall rating =1 weak sub-ratings; weak overall rating = ≥2 weak sub-ratings. | | | | | | | |

# Supplementary Table 3. Study and sample characteristics.

| Author, year, country | Study design, length | Sample size and participants | Age, mean (SD) | Gender (n, % female) | Outcomes | Adverse events |
| --- | --- | --- | --- | --- | --- | --- |
| Bickmore, 2013, USA | RCT, 12 m | n=263 older adults aged 65+ | 71.3 (5.4) | n=161 (61.2%) | Physical activity (International Physical Activity Questionnaire, Steps walked: Omron HJ-720ITC pedometers  Dietary behaviour (NIH/NCI Fruit and Vegetable Scan) | n=289 adverse events (n=8 control and n=2 intervention, mild- to moderate-severity events  related to participation in the study) |
| Bickmore 2013B, USA | RCT, 8 w | n=122 adults | Activity: 33.5 (12.8)  Diet: 32.9 (11.1)  Activity+Diet: 32.4 (12.3)  Control: 32.0 (14.5) | Activity: 16 (51.6%)  Diet: 17 (56.6%)  Activity+Diet: 22 (73.3%)  Control: 19 (61.3%) | Physical activity (steps, pedometer)  Dietary behaviour (NIH/NCI Fruit and Vegetable Scan) | None |
| Carfora, 2019, Italy | Panel design, 2 w | n=180 young adults | 20 (2) | n=136 (76%) | Red and processed meat consumption (food diary) | None |
| Carlin, 2021, United Kingdom | RCT, 12 w | n=25 families, with at least one child aged 5-12 years | Phase 1: Parents: 40.5 (5.4); Children: 9.1 (2.0)  Phase 2: Parents: 38.9 (5.2); Children: 7.9 (2.0) | Phase 1: Parents: 10 (91%); Children: 9 (56%)  Phase 2: Parents: 11 (73%); Children: 8 (44%) | Physical activity (ActiGraph GT3 accelerometer) | None |
| Cushing, 2021, USA | Non-RT, 20 d | n=40 adolescents aged 13-18 | Intervention: 15.25 (1.80)  Control: 15.25 (1.62) | Intervention: 15 (75%)  Control: 15 (75%) | Physical activity (ActiGraph wGT3X-BT) | None |
| De-Jongh González, 2022, Canada | RCT, 12 w | n=214 parent-child dyads | Parents: 44 (SD); children: 13 (SD 2.2) | Children: n=110 (51.4%) | Dietary behaviour (Waterloo Eating Behavior Questionnaire and the Canadian healthy Heating Index used as a measure of overall adherence to the 2007 Canada's Food Guide)  Physical activity (Fitbit Flex 2, Physical Activity Questionnaire Short Form) | None |
| Dhinagaran, 2021, Singapore | Pre-post, 4 w | n=60 adults | 33.7 (9.3) | n=37 (62%) | Physical activity (International Physical Activity Questionnaire)  Sleep (Pittsburgh Sleep Quality Index) | None |
| Friederichs, 2014, Netherlands | RCT, 4 w | n=958 adults 18-70 years old | 42.9 (14.5) | n=579 (60.4%) | Physical activity (Dutch Short Questionnaire to Assess Health Enhancing Physical Activity (SQUASH)) | None |
| Gardiner, 2017, USA | RCT, 30 d | n=61 women aged 18-50 | Intervention: 33 (8.1)  Control: 37 (8.4) | Overall: n=61 (100%) | Dietary behaviour (National Health and Nutrition Examination Survey (NHANES))  Physical activity (Stanford Patient Education Research Centre’s Exercise Behaviours Questionnaire) | None |
| Hassoon, 2021, USA | RCT, 4 w | n=45 adult cancer survivors | My Coach: 63.9 (9.3)  Smart Text: 64.1 (7.2)  Control: 58.1 (11.8) | My Coach: n=14 (100%)  Smart Text: n=11 (79%)  Control: n=13 (93%) | Physical activity (Steps, Objectively assessed) | None |
| King 2007, USA | RCT, 12 m | n=75 inactive adults aged 55+ | Human intervention: 60.5 (6.0)  Automated intervention: 61.6 (5.9)  Control: 60.2 (4.5) | Human advice intervention: n=47 (70.5%)  Automated advice intervention: n=43 (69.7%)  Control: n=42 (67.7%) | Physical activity (Stanford 7-day physical activity recall) | None |
| Kramer, 2020, Switzerland | Pre-post, 6 w | n=274 members of a health insurance company | 41.73 (13.54) | n=158 (57.66%) | Physical activity (Steps, smartphone app) | None |
| Lorenz 2019, Germany | RCT, 6 w | n=56 adults aged 18+ with insomnia | Intervention: 41.72 (17.31)  Control: 44.04 (20.05) | Intervention: 21 (72%)  Control: 18 (67%) | Sleep (Insomnia Severity Index) | None |
| Maher, 2020, Australia | Pre-post, 12 w | n=31 adults aged 45 to 75 | 56.2 (8.0) | n=21 (68%) | Physical activity (Active Australia Survey)  Dietary behaviour (14-item Australian Mediterranean diet adherence tool,  adapted from the Prevención con Dieta Mediterránea) | None |
| Phillip, 2022, France | Non-RT, 17 d | n=842 adults aged 18+ | Sleep Diary: 47.0 (13.6)  Kanopee: 51.2 (13.4) | Sleep Diary: n=339 (69.3%)  KANOPEE: 343 (64.1%) | Sleep (Insomnia Severity Index) | None |
| To, 2021, Australia | Pre-post, 6 w | n=151 physically inactive adults | Mean (SD) = 49.1 (9.3) | n=95 (81.9%) female | Physical activity, including steps (Fitbit flex, Active Australia survey) | None |
| Watson 2012, USA | RCT, 12 w | n=70 adults with BMI 25-35 kg/m^2^ | Intervention: 44.1 (SD NR)  Control: 40.6 (SD NR) | Intervention: 28 (80%)  Control: 31 (89%) | Physical activity (steps, ActiPed) | None |
| Werner-Seidler, 2019, Australia | Pre-post, 6 w | n=50 children aged 12–16 years with sleep difficulties. | 13.71 (1.35) | n=33 (66%) | Sleep (Insomnia Severity Index, Pittsburgh Sleep Quality Index, 10-item Sleep Diary) | None |
| Wright, 2013, USA | RCT, 12 w | n=50 parent-child dyads (child 9-12 years, BMI >95^th^ percentile) | Children (overall): 10.3 (1.1)  Parents (overall): 40.0 (9.1) | Children (overall): n=21 (42%)  Parents (overall): n=48 (96%) | Dietary behaviour (Block Dietary Data Systems Kids Food Screener version 2) | None |

# Supplementary Table 4. Intervention and chatbot characteristics.

| Author, year, country | Intervention overview | Intervention  duration and  frequency and length | Chatbot only  or multiple  components  intervention  2 | Target behaviour/s | Chatbot name, delivery method, and/or delivery platform | Output | Theoretical  framework | Behaviour change techniques (as reported in the paper) | Individualised |
| --- | --- | --- | --- | --- | --- | --- | --- | --- | --- |
| Bickmore, 2013, USA | Embodied Conversational  Agent (ECA) to motivate participants to increase walking. | Daily conversations with the ECA for 2 months | Multicomponent (chatbot and pedometer) | Physical activity | Embodied Conversational  Agent, software,  tablet | Voice, images (animated computer characters with voice, hand gesture, gaze cues, and  other nonverbal behaviour) | No | Review goals, positive reinforcement, identifying and problem-solving barriers, goal setting, | Yes |
| Bickmore 2013B, USA | Animated conversational agent to promote  physical activity and fruit and vegetable consumption | Daily | Multicomponent (chatbot and pedometer) | Physical activity Healthy eating | Animated conversational agent, computer | Text | No | Goal setting, develop and review goals, problem solving and identify and overcome barriers, increase knowledge of health behaviour | No |
| Carfora, 2019, Italy | Chatbot-based intervention to reduce red meat consumption. | Daily for 2 weeks | Chatbot only | Healthy eating (red meat consumption) | Facebook Messenger, smartphone | Text | No | Negative consequences on health | No |
| Carlin, 2021, United Kingdom | Intelligent personal assistant for promoting and maintaining physical activity and health behaviours | Daily and weekly tasks, prompts, and reminders for family members. | Chatbot only | Physical activity, healthy eating | Amazon Alexa, Amazon Echo Dot Smart speaker | Voice | No | None | Yes |
| Cushing, 2021, USA | Computer program  that adapts text messages based on user input to promote adolescent physical activity. | Multiple daily messages/ reminders | Chatbot only | Physical activity, sedentary behaviour | NUDGE, server-side computer program  that adapts text messages, smartphone | Text | No | Goal setting and review, self-monitoring, feedback  on goal attainment and revise future goals. | Yes |
| De-Jongh González, 2022, | App aimed to promote healthy lifestyle behaviours  by targeting dietary, physical  activity, screen time, and sleep. | NR | Chatbot only (as part of the app) | Physical activity, diet, screen time, sleep" | Aim2Be app (Ayogo Health Inc), Aimbot app, computer or smart phone with internet access | Text | Social cognitive theory, Player Experience  and Need Satisfaction Model, the Agency, Challenge,  Uncertainty, Discovery, and Outcomes framework | Self-regulatory strategies, goal setting, self-monitoring, and graded tasks to facilitate behaviour change by strengthening self-regulatory skills, enjoyment,  engagement, motivation, peer support, behaviour modeling,  interaction, social support | Yes |
| Dhinagaran, 2021, Singapore | A conversational agent promoting healthy lifestyle behaviour changes in the general population | Messages were sent to the participants four times a week (once for each of the 4 topics of focus) for 4 weeks | Chatbot only | Physical activity, healthy eating, sleep | Chatfuel (Precilla), a Facebook Messenger smartphone | Text | Capability, Opportunity, Motivation,  Behavior model | None | Yes |
| Friederichs, 2014, Netherlands | Web-based, motivational interviewing intervention with an avatar, to increase physical activity | NR | Chatbot only | Physical activity | Computer | Avatar, text | No | Motivational interviewing | No |
| Gardiner, 2017, USA | Embodied Conversational Agent (ECA) to teach lifestyle modifications to urban women. | Daily for 30 days | Chatbot only | Physical activity, healthy eating, mindfulness, stress management) | Gabby embodied conversational agent; web-based, computer | Text, images, speech | No | Goal setting, problem solving, education | Yes |
| Hassoon, 2021, USA | AI-based health  coaching agent (MyCoach) delivered through a smart speaker or AI-based autonomous progressive smart coaching delivered through  text messaging (SmartText) | Daily | Multi-component (smart speaker or SMS chatbot, Fitbit Charge HR2) | Physical activity | MyCoach (Amazon Echo/  Alexa smart speaker); MyText, (smartphone) | Voice  or text | NR | NR | Yes |
| King 2007, USA | Telephone-assisted physical activity counselling by an automated telephone-linked computer system | 15x 10-15 min contacts during the study | Multi-component (telephone-linked computer system, physical activity mailouts, pedometer) | Physical activity | CHAT "Community Health Advice by Telephone", conversational agent, telephone-linked computer system | Voice | Social Cognitive  Theory and the Transtheoretical Model | Physical activity assessment,  progress evaluation, problem-solving,  goal setting, feedback, positive support and tailored  advice, self-monitoring. | Yes |
| Kramer, 2020, Switzerland | Chatbot-guided interventions to  encourage users to reach personalized daily step goals. | Every day, the Ally app sets a personalized activity  goal based on each participant’s past activity data. | Smartphone app (with chatbot included) | Physical activity | Ally, smartphone app, | Text | Health Action Process  Approach and self-determination theory | Incentives, Planning, Self-monitoring prompts, Goal setting | Yes |
| Lorenz 2019, Germany | Online intervention with automated feedback for treatment of insomnia. | 6 sessions of  CBT-I with an animated sleep coach | Chatbot only | Sleep | Mementor Somnium, internet-based (computer) | Speech, visual (graphs) | CBT-I | Goal setting, intention formation,  barrier identification, problem solving, feedback, review of goals,  information on consequences, action planning, instruction, prompts/ cues | Yes |
| Maher, 2020, Australia | PA and diet intervention (MedLiPal) delivered via artificially intelligent virtual health coach. | 11 weekly check-ins and 24/7 chatbot | Multi-component (Paola, Garmin wearable activity monitor, MedLiPal website, diet and activity log) | Physical activity  Healthy eating | Paola, iOS or Android smartphone or tablet; Messaging app (Slack) | Text | None | Goal setting, Problem-solving, Goal review, Self-monitoring with feedback, Social support, Reattribution, Use of credible sources | Yes |
| Phillip, 2022, France | Smartphone-Based Virtual Companion to Treat  Insomniac Complaints | Daily | Chatbot only | Sleep | Louise, Kanopee smartphone app, | Text, graphs/ figures, images | No | Feedback | Yes |
| To, 2021, Australia | Machine learning–based physical  activity chatbot that sent out daily motivational messages in relation to goal achievement, and automatically adjusted the daily goals | Daily updates on the physical activity level for self-monitoring, sent out daily motivational messages | Multi-component (Chatbot + Fitbit) | Physical activity | Ida, The Facebook Messenger app, Smartphone | Text | COM-B model | Capability (adaptive feedback on goal achievement), Opportunity (educational content), and motivation, goal setting, self-monitoring. | Yes |
| Watson 2012, USA | Virtual coach (automated and follows an algorithm-driven script) to increase physical activity | 3+ times a week for 5-10 minutes each session. | Multi-component (Chatbot, pedometer, ActiHealth website) | Physical activity | Virtual coach, (conversation agent), software installed on home computer | Speech, text, images | Behavioural and social cognitive theory | Goal setting, shaping, self-monitoring, positive reinforcement, problem solving, education, and social support. | Yes |
| Werner-Seidler, 2019, Australia | CBT-based Sleep ninja app (with integrated chatbot) for insomnia | 6x 5-10min training sessions and nightly prompts | Chatbot only | Sleep | Sleep ninja app,  smartphone running iOS or  Android | Text and visual images | No | Stimulus control | Yes |
| Wright, 2013, USA | Automated interactive  voice response system, to deliver a healthy weight management and sedentary behaviour intervention. | Twice weekly for 12 weeks | Chatbot only | Healthy eating, physical activity, sedentary behaviour (TV time) | Automated interactive  voice response (IVR) systems, telephone | Voice | Social Cognitive Theory | Goal setting, contracting, parent-child meetings, problem-solving, self-monitoring, instructions on how to perform the behaviour | Yes |

# Supplementary Table 5. Subgroup analyses (using data from randomised controlled trials and single-group pre-post studies) for total physical activity, steps and fruit and vegetable consumption.

|  | Number of studies | Number of participants | I^2^ (%) | Standardised mean difference (95% CI) | P-value |
| --- | --- | --- | --- | --- | --- |
| Total physical activity |  |  |  |  |  |
| *Intervention duration* |  |  |  |  | 0.83^1^ |
| ≤6 weeks | 4 | 882 | 21 | 0.29 (0.13, 0.46) | <0.01 |
| >6 weeks | 6 | 661 | 15 | 0.27 (0.08, 0.45) | <0.01 |
| *Intervention type* |  |  |  |  | 0.81^1^ |
| Chatbot only | 5 | 934 | 49 | 0.27 (0.03, 0.51) | 0.03 |
| Multicomponent intervention | 5 | 493 | 0 | 0.31 (0.14, 0.47) | <0.01 |
| *Output* |  |  |  |  | 0.88^1^ |
| Speech/voice | 4 | 477 | 45 | 0.32 (0.02, 0.63) | 0.04 |
| Text | 6 | 1126 | 0 | 0.30 (0.18, 0.42) | <0.01 |
| *Assessment method* |  |  |  |  |  |
| Self-report | 3 | 305 | 0 | 0.29 (-0.02, 0.61) | 0.07 |
| Objective measure | 7 | 1298 | 10 | 0.28 (0.15, 0.41) | <0.01 |
| *Use of AI/NLP* |  |  |  |  | 0.57^1^ |
| Yes | 6 | 600 | 16 | 0.24 (0.04, 0.44) | 0.02 |
| No | 4 | 1003 | 16 | 0.31 (0.17, 0.46) | <0.01 |
| Steps |  |  |  |  |  |
| *Intervention duration* |  |  |  |  | 0.08^1^ |
| ≤6 weeks | 3 | 706 | 0 | 0.36 (0.22, 0.49) | <0.01 |
| >6 weeks | 3 | 454 | 0 | 0.14 (-0.05, 0.33) | 0.14 |
| *Intervention type* |  |  |  |  | 0.66^1^ |
| Chatbot only | 3 | 862 | 34 | 0.30 (0.09, 0.51) | <0.01 |
| Multicomponent intervention | 3 | 308 | 0 | 0.23 (0.04, 0.43) | 0.02 |
| *Output* |  |  |  |  | 0.23^1^ |
| Speech/voice | 2 | 332 | 0 | 0.17 (-0.05, 0.39) | 0.12 |
| Text | 4 | 944 | 0 | 0.32 (0.19, 0.46) | <0.01 |
| *Use of AI/NLP* |  |  |  |  | 0.79^1^ |
| Yes | 3 | 396 | 0 | 0.25 (0.04, 0.45) | 0.02 |
| No | 3 | 880 | 33 | 0.28 (0.10, 0.46) | <0.01 |
| Fruit and vegetable consumption |  |  |  |  |  |
| *Intervention duration* |  |  |  |  | 0.17 |
| ≤6 weeks | 2 | 83 | 63 | 0.16 (-0.63, 0.96) | 0.68 |
| >6 weeks | 3 | 186 | 9 | 0.77 (0.45, 1.09) | <0.01 |
| *Intervention type* |  |  |  |  | 0.79 |
| Chatbot only | 1 | 61 | NA | 0.51 (0.00, 1.02) | 0.05 |
| Multicomponent intervention | 3 | 228 | 50 | 0.59 (0.25, 0.93) | <0.01 |
| *Output* |  |  |  |  | 0.02^1^ |
| Speech/voice | 2 | 105 | 34 | 0.16 (-0.34, 0.66) | 0.53 |
| Text | 2 | 184 | 0 | 0.90 (0.57, 1.23) | <0.01 |
| *Use of AI/NLP* |  |  |  |  | 0.02^1^ |
| Yes | 2 | 184 | 0 | 0.90 (0.57, 1.23) | <0.01 |
| No | 2 | 105 | 34 | 0.16 (-0.34, 0.66) | 0.53 |
| Sleep duration |  |  |  |  |  |
| *Intervention type* |  |  |  |  | 0.04^1^ |
| Chatbot only | 1 | 56 | NA | 0.48 (-0.05, 1.01) | 0.08 |
| Multicomponent intervention | 2 | 1168 | 97 | 1.45 (-0.61, 3.51) | 0.17 |
| Sleep quality |  |  |  |  |  |
| *Intervention type* |  |  |  |  | 0.02^1^ |
| Chatbot only | 2 | 116 | 86 | 0.41 (-0.48, 1.30) | 0.37 |
| Multicomponent intervention | 2 | 1168 | 95 | 0.96 (-0.43, 2.35) | 0.17 |
| ^1^ P-value represents overall test for subgroup differences (Hedges g).  AI: artificial intelligence; NLP: natural language processing. | | | | | |


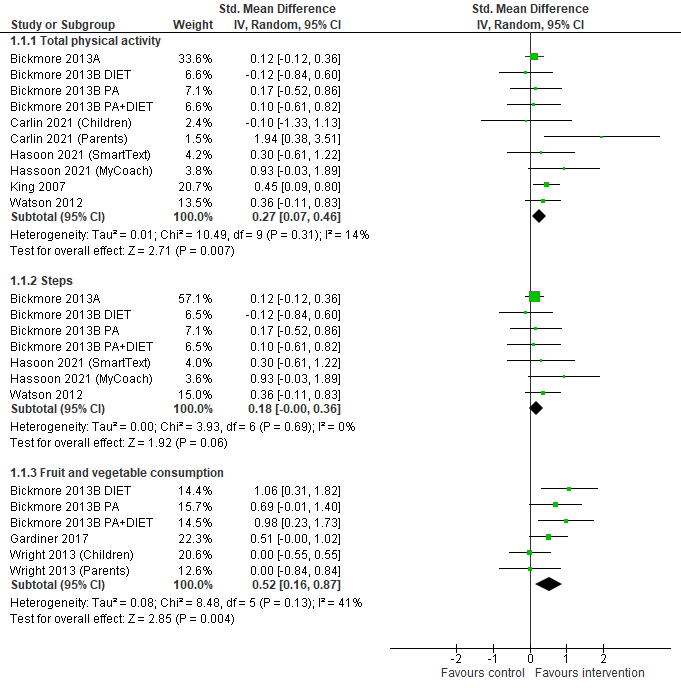


# Supplementary Figure 1. Meta-analyses of effects of chatbot intervention versus control conditions (using data from randomised controlled trials only) for overall physical activity, steps, and fruit and vegetable consumption (there was insufficient data for all other outcomes to conduct meta-analyses) using Hedge’s g.
